# Threat vigilance and intrinsic amygdala connectivity

**DOI:** 10.1002/hbm.25851

**Published:** 2022-04-01

**Authors:** Peter A. Kirk, Avram J. Holmes, Oliver J. Robinson

**Affiliations:** ^1^ Institute of Cognitive Neuroscience University College London London UK; ^2^ Experimental Psychology University College London London UK; ^3^ Departments of Psychology and Psychiatry Yale University New Haven Connecticut USA; ^4^ Wu Tsai Institute Yale University New Haven Connecticut USA; ^5^ Clinical, Educational and Health Psychology University College London London UK

**Keywords:** amygdala, anxiety, fMRI, subcortex

## Abstract

A well‐documented amygdala‐dorsomedial prefrontal circuit is theorized to promote attention to threat (“threat vigilance”). Prior research has implicated a relationship between individual differences in trait anxiety/vigilance, engagement of this circuitry, and anxiogenic features of the environment (e.g., through threat‐of‐shock and movie‐watching). In the present study, we predicted that—for those scoring high in self‐reported anxiety and a behavioral measure of threat vigilance—this circuitry is chronically engaged, even in the absence of anxiogenic stimuli. Our analyses of resting‐state fMRI data (*N* = 639) did not, however, provide evidence for such a relationship. Nevertheless, in our planned exploratory analyses, we saw a relationship between threat vigilance behavior (but not self‐reported anxiety) and intrinsic amygdala‐periaqueductal gray connectivity. Here, we suggest this subcortical circuitry may be chronically engaged in hypervigilant individuals, but that amygdala‐prefrontal circuitry may only be engaged in response to anxiogenic stimuli.

## 1 INTRODUCTION

A growing body of literature has outlined a cortico‐subcortical network which is engaged during states of anxiety. A core feature of this network appears to be amygdala‐dorsomedial prefrontal circuitry (Milad et al., 2007; Roy et al., 2013; Vidal‐Gonzalez et al., 2006), which has been theorized to drive attentional amplification of threat‐relevant stimuli in the environment (Robinson et al., 2012; Robinson et al., 2013). Individual differences in attention to threat (“threat vigilance”) are thought to be a key feature driving variation in trait anxiety (MacLeod & Mathews, 1988; Grupe & Nitschke, 2013). Indeed, hyper‐engagement of this amygdala‐prefrontal circuit while under induced anxiety has been observed in clinically anxious populations (Robinson et al. 2014). As such, the study of amygdala‐prefrontal circuitry has been a primary line‐of‐inquiry in the anxiety literature. However, there is likely a significantly wider network beyond this core circuit.

The bed nucleus of the stria terminalis (BNST), a region dorsal to‐ and highly connected with the amygdala is also thought to play a core role in coordinating adaptive responses to potential dangers (Hur et al., 2020). Early work suggested that—while the amygdala was thought to react to immediate threats (fear responses)—the BNST was associated with processing chronic, uncertain threats (anxious responding; Davis, 2006). However, this framework remains disputed (Fox & Shackman, 2019). Nonetheless, the BNST, also known as the “extended amygdala”, has an established role in processing ambiguous threats and, consequently, is heavily implicated in anxiety (Hur et al., 2020). Additionally, other subcortical regions such as the hypothalamus and periaqueductal gray may form a key junction between threat‐relevant perceptual/cognitive processes and the embodied responses associated with anxiety: engagement of fight‐flight‐freeze behaviors and alterations in autonomic functioning (Deng et al., 2016).

A breadth of research has demonstrated top‐down, cortico‐subcortical projections. In addition to the dorsomedial prefrontal cortex, other regions in the cortex have been implicated, such as: the anterior insula, associated with a range of functioning including anxiety‐relevant interoceptive sensitivity, anticipation of future events, and controllability of stressors (Terasawa et al., 2013; Grupe & Nitschke 2013; Limbachia et al., 2021); subgenual anterior cingulate, related to threat‐relevant memory processes (Hakamata et al., 2020); and an anterior section of the ventromedial prefrontal cortex/medial orbitofrontal cortex (Pujara et al., 2019), which may relate to positive affect and/or safety signal integration (Myers‐Schulz & Koenigs, 2012; Tashjian et al., 2021), though relatively less attention has been paid to its functional distinction from subgenual ACC. Broadly speaking, projections from the cortex have typically been thought of as providing regulatory, evaluative, and contextual inputs to fundamental threat processes in subcortical regions (Tillman et al., 2018). While the aforementioned regions have been selectively associated with specific perceptual/cognitive processes, these likely operate as a broad, interactive network to orchestrate defensive behaviors (Gorka et al. 2018; Chavanne & Robinson, 2021).

We have now seen a plethora of anxiety research implicating this “defensive response network” spanning cortical and subcortical regions (Abend et al., 2022). However, despite a wide range of research establishing an association between anxiety and this network, our understanding of individual differences in this circuitry nonetheless remains limited. There is substantial literature establishing the presence of amygdala‐dorsomedial prefrontal connectivity while under induced anxiety, but designs are not typically powered for large‐scale individual differences research. On the other hand, large‐scale resting‐state studies sometimes include such circuitry within multivariate models, which have started to emerge as useful in the prediction of self‐reported anxiety (Li et al., 2019). However, there can be difficulties interpreting the contributions of feature weights in these models (e.g., nonlinear support vector machines; Misaki et al., 2010) and how they relate to true brain activity/connectivity. Thus, model parameters often do not directly grant access to physiological information and necessitate transformations before attempting to estimate this information (Haufe et al., 2014). Thus, with the frequent goal of behavioral prediction, these studies often focus on models' decoding accuracy of psychiatric symptoms; consequently, there is often less of a focus on elucidating low‐level mechanisms associated with this circuitry. Among resting‐state research that has included amygdala‐prefrontal circuitry as a focal point, there is little consensus. For individuals with clinical anxiety, multiple studies have demonstrated both increased and aberrant amygdala‐prefrontal connectivity at rest (see Mizzi et al., 2021). Despite accelerated developments in analytical tools, much of this research has remained dependent on diagnostic criteria and/or self‐report measures which may have high underlying heterogeneity (Cuthbert & Insel, 2013). Secondly, as these measures depend on introspection, they may not be comparable across individuals nor tap into precise internal processes (Baumeister et al., 2007; Watson et al., 2017). Moreover, individual scales are often associated with differing and/or multiple latent factors (Rose & Devine, 2014). While these measures may be a useful tool to implicate whether regions and connections are *generally* implicated in anxiety, they provide little theoretical precision as to the processes underlying brain circuitry.

In a recent analysis, we demonstrated that individual differences in anxiety were associated with amygdala‐dorsomedial prefrontal dynamics during movie‐watching (Kirk et al., 2021); however, effects were notably stronger for a targeted behavioral measure of threat vigilance than self‐reported anxiety. We noted that subjects' reaction time/accuracy to fearful faces correlated slightly higher with suspense and amygdala‐prefrontal dynamics (*r* = −.19) than did self‐reported scores from the Hospital Anxiety and Depression Scale (*r* = −.16). This supports the notion that perceptual/attentional processes related to threat are a key function of this circuitry. Counter to our predictions, we saw effects primarily during low suspense scenes. As visualizations demonstrated the possibility of a more nuanced, dynamic relationship between anxiety, connectivity, and suspense than that indicated by linear correlation, we offered three interpretations for these effects: (1) high trait anxiety individuals chronically engage amygdala‐prefrontal threat circuitry *irrespective of the anxiogenic features of the environment*; (2) high trait anxiety individuals show greater apprehension of anxiogenic scenes; or (3) anxiety slows the habituation of threat circuitry following anxiogenic scenes. In order to explore this “chronic engagement” hypothesis of amygdala‐prefrontal connectivity, we sought to test whether anxiety was associated with functional connectivity in the same individuals, but during resting‐state scanning. Specifically, we investigated whether this relationship was apparent for “intrinsic functional connectivity” of the amygdala and dorsomedial prefrontal connectivity as derived from eyes‐closed resting‐state scans in a separate imaging run, but in the *same* subjects using the *same* anxiety measures. In planned exploratory analyses, we also sought to test whether intrinsic connectivity of a wider defensive response network was associated with anxiety measures.

### 1.1 Hypotheses

We made the following key predictions regarding a resting‐state fMRI dataset. Each tested left and right amygdala connectivity separately and were preregistered on the Open Science Framework (https://osf.io/cfdq7/):

Self‐reported anxiety will positively correlate with intrinsic amygdala‐dorsomedial prefrontal connectivity.

A behavioral measure of threat vigilance will positively correlate with intrinsic amygdala‐dorsomedial prefrontal connectivity.

## 2 METHOD

The present resting‐state project was conducted following a related analysis on movie‐watching data. Given the similarity in methods, wording here may overlap with that from the prior manuscript (Kirk et al., 2021). Experiment code and data derivatives are available at the Open Science Foundation (https://osf.io/qzmgb/). Raw data are available on request from the Cam‐CAN website (https://camcan-archive.mrc-cbu.cam.ac.uk/dataaccess/).

### 2.1 CamCAN dataset

#### 2.1.1 fMRI data

We conducted analyses on the Cambridge Centre for Ageing Neuroscience database (CamCAN; N = 652, age mean = 54.81, age SD = 8.54, age range = 18.5–88.9, 330 female, 320 male, 50/589 left/right handed, 11 ambidextrous, 2 missing hand data; Shafto et al., 2014; Taylor et al., 2017). The present study made use of volumes acquired during eyes‐closed resting‐state scanning. BOLD signal was acquired with a T2* GE EPI (32 axial slices 3.7 mm thick, 0.74 mm gap, TR = 1970 ms; TE = 30 ms, FA = 78 deg; FOV = 192 mm × 192 mm; 3 × 3 × 4.44 mm, TA = 8 min 40 s). The functional data were already preprocessed using the following steps: realignment and unwarping with field maps, slice‐time correction, transformation to MNI space, and despiking using outlying wavelet coefficients (no smoothing). For a full overview of database details, see Taylor et al. (2017).

#### 2.1.2 Self‐report/behavioral data

Prior to scanning, participants completed the Hospital Anxiety and Depression Scale (HADS; Zigmond & Snaith, 1983). The anxiety section of this scale (7 items; Cronbach's *α* = ~.83; Bjelland, 2002) constituted our self‐report metric for our first hypothesis. Subjects with no available HADS data (*N* = 3) were omitted from the relevant analyses, leaving 649 participants (mean/SD of anxiety scores = 4.96/3.30).

We also previously conducted analyses on whether a behavioral measure of attentional bias to threat (“threat vigilance”) also correlated with connectivity. For this, we calculated threat vigilance measures from a face perception task participants completed prior to scanning (“emotion expression recognition”). This included labeling faces morphed between emotional expressions (happiness‐surprise, surprise‐fear, fear‐sadness, sadness‐disgust, disgust‐anger, anger‐happiness; stimuli derived from Ekman & Friesen, 1976). Our threat vigilance measure was calculated through drift diffusion modeling of fearful facial expressions. Our choice for the use of fearful faces was based on the notion that these signal uncertain threats, a feature of the environment typically associated with anxiety/vigilance (Mobbs et al., 2020).

**FIGURE 1:**
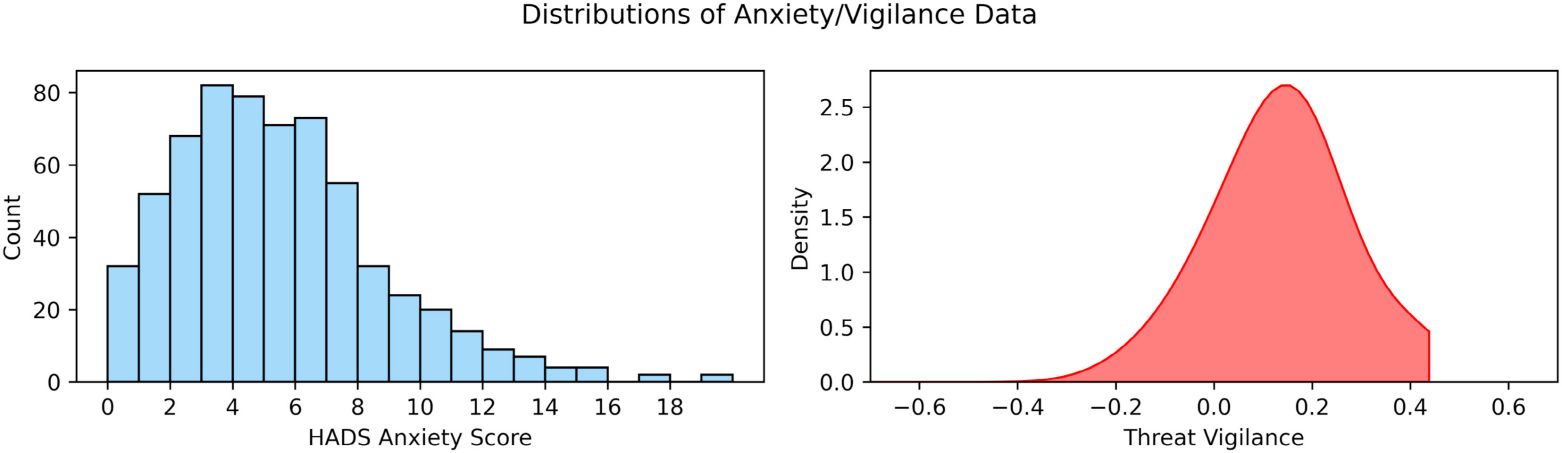
Kernel density plots of self–reported HADS anxiety and threat vigilance measures (visualized with Seaborn and matplotlib; Waskom, 2021; Hunter, 2007). Due to low accuracy in the facial emotion task, 5 subjects had extremely low drift rate parameters of −3.1 (not visualized, but retained in analyses)

We first extracted accuracy and mean/variance of RT for correctly labeled trails where morphs contained 70/90% fear (summary statistics used as trial‐by‐trial data for each morph step are not provided within the CamCAN dataset). These were then inputted into E–Z drift‐diffusion modeling (Wagenmakers et al., 2007). The drift parameter constituted our threat vigilance metric. RT variance values of 0 (one correct trial) and accuracy values of 0, 0.5, and 1 were increased (or decreased in the last case) by .000001 to avoid division errors. Subjects with no available face data (*N* = 15) were omitted from the relevant analyses. We previously reported a small but significant positive relationship between self‐reported anxiety and threat vigilance measures (*ρ* = .13, *p* = .0008; Kirk et al., 2021; distributions plotted in Figure 1).

### 2.2 Analysis

fMRI time series extraction and modeling was conducted using AFNI (Cox, 1996) and Python. Relevant functions are denoted in parentheses. All analyses used two‐sided tests thresholded at *α* = .05 unless otherwise stated. All tests were preregistered (https://osf.io/cfdq7/). Deviations to preregistration were: repeating analyses on data following global signal regression (see *within subject modeling*; we report below which tests were conducted post hoc); providing complementary approximate Bayes Factors as estimated from *p* values (Wagenmakers, 2022); and a supplementary mediation analysis (see Data S1).

#### 2.2.1 Regions of interest masks

**FIGURE 2:**
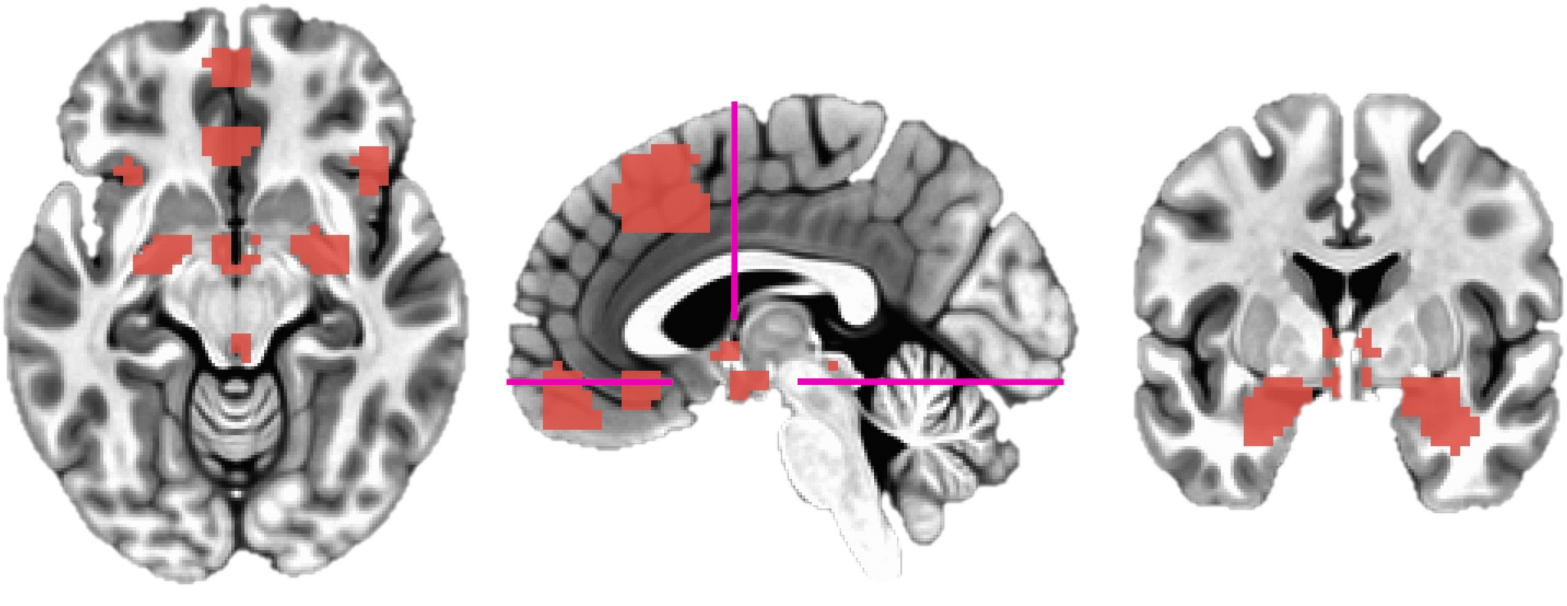
ROI definitions. Left: axial view showing anterior vmPFC, sgACC, anterior insula, hypothalamus (80% Freesurfer overlap), amygdala (dilated MNI mask used for constraining segmentations), and periaqueductal gray masks. Middle: sagittal view showing our dmPFC mask; pink lines refer to axial and coronal slices. Right: coronal view showing BNST, hypothalamus, and dilated amygdala masks

For hypothesis‐testing, our key regions of interest were the amygdala and dorsomedial prefrontal cortex: our amygdala ROIs were selected from previously generated anatomical parcellations of T1 images in Freesurfer (Fischl, 2012) constrained within a dilated MNI amygdala mask (Kirk et al., 2021; Tzourio‐Mazoyer et al., 2002); our dmPFC mask was selected from a meta‐analysis demonstrating the conjunction of adaptive/maladaptive anxiety (“induced (+) vs. transdiagnostic (+) 20 mm”, Chavanne & Robinson, 2021; https://neurovault.org/images/384691/; Figure 2). The latter mask was generated based on an overlap between activation‐based results contrasting unpredictable‐threat vs safe conditions and clinical vs healthy subjects (pooled across two or more anxiety disorders). This was chosen so as to be atheoretical regarding the distinction between the neural manifestation of adaptive and maladaptive anxiety.

For exploratory analyses, we were also interested in the hypothalamus, bed nucleus of the stria terminalis (BNST), periaqueductal gray (PAG), medial orbitofrontal cortex/anterior ventromedial prefrontal cortex (vmPFC), subgenual anterior cingulate cortex (ACC), and anterior insula. Subcortical structures were defined anatomically: BNST and periaqueductal gray masks were based on previous manual tracings (in MNI space) of 10 and 53 subjects, respectively (Theiss et al., 2017; Weis et al., 2021); and hypothalamus was defined through Freesurfer parcellations (Billot et al., 2020; all subunits combined due to the EPI voxel resolution). Cortical structures were defined functionally from meta‐analytic clusters (Chavanne & Robinson, 2021; Anterior Insula = “Induced (+) vs. Pathological anxiety (+) 20 mm”, anterior vmPFC = “Induced anxiety (threat > safe) 20 mm”; subgenual ACC = “transdiagnostic anxiety (patients > controls)”). Medial structures where lateralizations were neighboring (BNST, PAG, hypothalamus, dmPFC, anterior vmPFC, subgenual ACC) were collapsed bilaterally. Given the spatial resolution of scans and likelihood of minor misalignments, the BNST and periaqueductal gray masks were dilated (three times) and eroded (two times) in order to create masks which would overlap with more participants (though at the cost of potential unrelated noise/signal; see Section 4).

Participants with failed Freesurfer segmentations (*N* = 10) were excluded from analyses. Combined with missing self‐report/behavioral data, this left 642 participants for main‐effects tests (1.53% dropout), 639 participants for tests on self‐report measures (1.99% dropout), and 627 participants for tests on threat vigilance measures (3.83% dropout). We note here that nine participants who were not included in our previous movie‐watching analysis (due to issues of timeseries extraction from a canonical 400 parcel solution) were included in the present analysis.

#### 2.2.2 Within‐subject modeling

We first removed effects of no interest from our raw time series (“3dDeconvolve”) by regressing out baseline signals with drift [−polort A] (demeaning and detrending) and 24 motion parameters (raw + squares + temporal derivatives + derivatives squared) to produce a cleaned time series (we also highlight here that data the was previously despiked and we include motion parameters in our between‐subjects modeling). We then extracted ROI seeds (“3dmaskave”) from the cleaned time series. For each subject we then calculated (Fisher‐transformed) Pearson correlations between all ROIs.

As our original analysis did not remove global BOLD signals, this had the potential to mask anti‐correlated regions (Murphy & Fox, 2017). Following planned analyses, we generated a second preprocessed dataset (post hoc) by including a 25th, global signal regressor which was generated by taking the mean timeseries of all voxels within auto‐masked volumes. For visualization purposes, we also calculated whole‐brain, voxelwise amygdala correlations on this data (“3dTcorr1D”), which we projected onto a surface using NIlearn (Abraham et al., 2014).

#### 2.2.3 Group‐Level modeling

For group‐level tests we first looked at average within‐subject connectivity via *t*‐tests for all pairs of ROIs. For between‐subjects effects, we conducted partial spearman correlations between anxiety measures (self‐report/threat vigilance) and amygdala‐dmPFC connectivity, including age, sex, and motion (mean framewise displacement) as covariates of no interest. All tests were reconducted post hoc on data which had been preprocessed with global signal regression. We report uncorrected results and those which surpass Bonferroni correction across 45 edges (*p* < .0011).

## 3 RESULTS

### 3.1 Average connectivity

**FIGURE 3:**
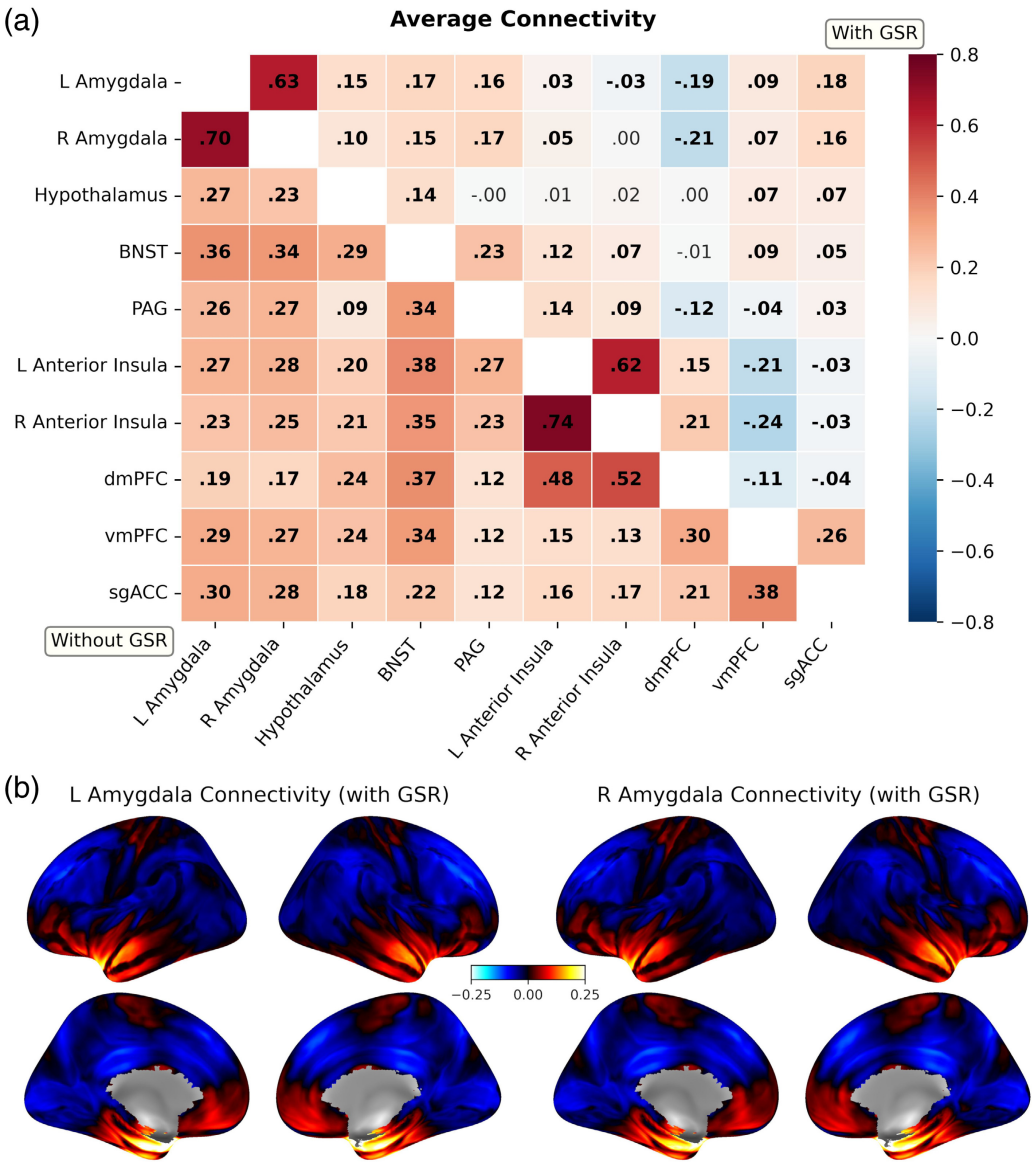
Average correlations of timeseries between all ROIs (“functional connectivity”). (a) Heatmap of average functional connections between regions before (lower triangle) and after (upper triangle) global signal regression (GSR). Displayed are non‐Fisher transformed r for visualization purposes. Bolded cells refer to connections significant at Bonferroni‐corrected *p* < .05. (b) Average amygdala‐whole brain functional connectivity (*r*) following global signal regression (no thresholding). BNST, Bed nucleus of the stria terminalis; PAG, periaqueductal gray; dmPFC, dorsomedial prefrontal cortex; vmPFC, (anterior) ventromedial prefrontal cortex; sgACC, subgenual anterior cingulate cortex

In our original, planned analysis, all regions demonstrated positive functional connections and surpassed Bonferroni correction for all 45 edges [*t* (641) range = 11.3:110.8, *p* < .00001)]. Following global signal regression, the polarity of some of these connections was altered (Figure 3). Applying global signal regression had no impact on the direction or significance of inference for our subsequent, statistically corrected results.

### 3.2 Hypothesis testing

**FIGURE 4:**
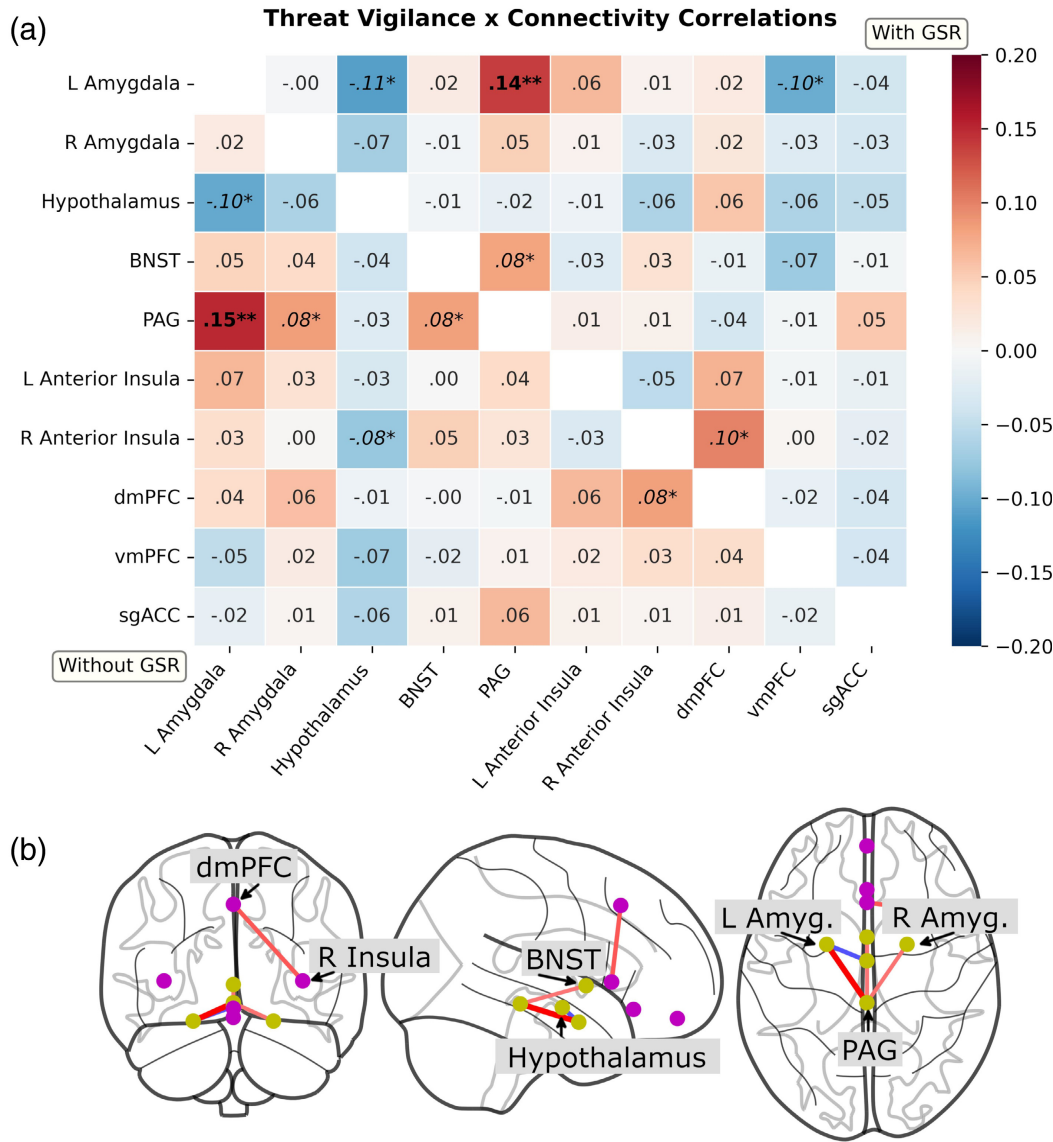
Partial spearman correlations between threat vigilance and functional connectivity (*ρ*; adjusting for age, sex, and mean motion). (a) Heatmap of correlations before (lower triangle) and after (upper triangle) global signal regression (GSR). **p < .05, Bonferroni‐corrected across 45 edges; *p < .05, uncorrected. Only the left amygdala‐periaqueductal gray connection survives correction regardless of GSR. (b) Glass brain plot of uncorrected correlations (*p* < .05 uncorrected with GSR). BNST, bed nucleus of the stria terminalis; PAG, periaqueductal gray; dmPFC, dorsomedial prefrontal cortex; vmPFC, (anterior) ventromedial prefrontal cortex; sgACC, subgenual anterior cingulate cortex

To test our key hypotheses, we conducted partial spearman correlations between anxiety measures and amygdala‐dmPFC connectivity, including age, sex, and motion (mean framewise displacement) as covariates of no interest. Self‐reported anxiety did not demonstrate a significant relationship with amygdala‐dmPFC connectivity [left amygdala: *ρ* = .004, *p* = .90, BF_01_ = 4.78; right amygdala: *ρ* = .03, *p* = .51, BF_01_ = 3.90]. Threat vigilance also did not demonstrate a significant relationship [left amygdala: *ρ* = .04, *p* = .37, BF_01_ = 3.04; right amygdala: *ρ* = .06, *p* = .13, BF_01_ = 4.14; Figure 4].

### 3.3 Exploratory tests

For planned exploratory analyses, we repeated partial spearman correlations between anxiety measures and all pairs of ROIs, applying Bonferroni correction for all 45 edges (*p* < .0011). Self‐reported anxiety was not significantly related to any of our connectivity measures. However, threat vigilance was significantly associated with increased left amygdala‐periaqueductal gray functional connectivity [*ρ* = .15, *p* = .0001, BF_01_ = 0.06]. We subsequently repeated our analyses post hoc on data which had been preprocessed with global signal regression. This did not alter our inference: threat vigilance again demonstrated a significant relationship with increased left amygdala‐periaqueductal gray functional connectivity [*ρ* = .14, *p* = .0005, BF_01_ = 0.11; Figure 4].

## 4 DISCUSSION

In the present study we sought to test whether intrinsic (resting‐state) functional connectivity of an amygdala‐dorsomedial prefrontal circuit, thought to underlie attention to threat, correlated with individual differences in anxiety. We looked at both self‐reported anxiety and a behavioral measure of attention to threat (“threat vigilance”), derived from the accuracy and reaction times of face perception data. Testing our key hypotheses, we did not observe a relationship between our anxiety measures and amygdala‐dorsomedial prefrontal connectivity. In our planned exploratory analyses, we did however observe a correlation between threat vigilance and heighted functional connectivity between the amygdala and periaqueductal gray.

Our hypothesis—that trait anxiety measures would positively correlate with intrinsic amygdala‐prefrontal connectivity—was motivated by threat‐of‐shock studies (Robinson et al., 2012), the resting‐state literature (which has shown mixed results, see Mizzi et al., 2021), and our previously observed results associating individual differences in anxiety and this circuitry to suspenseful dynamics during movie‐watching (Kirk et al., 2021). In the latter, we observed a negative correlation between anxiety‐relevant alterations in amygdala‐dmPFC connectivity and suspense: one interpretation of our findings was that—while low anxiety individuals selective engage this circuit in response to threat—highly anxious individuals chronically engage this circuit, irrespective of anxiogenic cues; another interpretation was that highly anxious individuals have slowed habituation and/or greater apprehension responses to anxiogenic scenes. If highly anxious individuals chronically engage this circuit even in the absence of salient stimuli, we would expect differences to also be apparent during resting‐state scanning.

Here, we report no significant relationship between self‐report/vigilance and amygdala‐dmPFC connectivity. This provides insight into how this circuitry is recruited across individuals. In line with the threat‐of‐shock literature, engagement of this circuitry may only arise in response to anxiogenic stimuli; consequently, individual differences are not observed in the absence of such perturbations (i.e., eyes‐closed resting‐state scanning). *Even if a true effect were present* in the dataset, we highlight here: (a) the relatively large sample size (*N* = 639); (b) lack of detecting these effects across two differing preprocessing pipelines; (c) inconsistent findings from prior resting‐state studies (Mizzi et al., 2021); (d) approximate Bayes Factors suggested moderate evidence in favor of the null; and (e) previously observed effects in the same subjects with the same masks during movie‐watching (Kirk et al., 2021). Consequently, we argue that any potential effect—if true—is of a theoretically negligible magnitude compared to effects following induced anxiety.

After hypothesis‐testing we conducted a series of planned exploratory tests on a wider “defensive response” network which included other subcortical and cortical regions implicated in anxiety and threat vigilance (Abend et al., 2022; Grupe & Nitschke 2013). Here, we detected a relationship between amygdala‐periaqueductal gray connectivity and threat vigilance (but not self‐report). Main effects tests suggest these were functionally positive connections that were heightened among those scoring high in threat vigilance. In other words, more behaviorally vigilant individuals demonstrated heightened positive amygdala‐PAG connectivity. These regions—and their connection—have been repeatedly implicated in top‐down, anxiety‐relevant regulation of autonomic functioning and fight‐flight‐freeze behaviors (for reviews, see Faull et al., 2019; Lefler et al., 2020).

Stimulation‐based work in rodents has demonstrated populations of neurons within the periaqueductal gray which respond to threat detection and threat responsive behaviors (Deng et al., 2016; Wang et al., 2021). In addition to typical evoked activation, primate research has demonstrated associations between individual differences in anxious temperament and functional connectivity between the amygdala and periaqueductal gray (Fox et al., 2018). Human fMRI work has demonstrated an association between evoked anxiety and periaqueductal gray activation (Mobbs et al., 2007; Hur et al., 2020). One study in humans using an anxiety induction did not find interactions between functional connectivity of the periaqueductal gray and the degree of evoked anxiety or clinical diagnosis (Abend et al., 2022). Here, we provide evidence that, unlike amygdala‐prefrontal connections, individual differences in amygdala‐periaqueductal circuitry may be apparent at rest; specifically, this circuitry may be chronically engaged in hypervigilant individuals, even in the absence of threatening cues/anxiogenic stimuli.

Prior resting‐state research contrasting clinical groups has failed to observe such associations with amygdala‐periaqueductal gray connectivity (Anteraper et al., 2014). We believe this is likely related to the different latent factors measured by self‐report scales and the functional processes underlying this subcortical circuitry. We previously noted only a small association between our self‐report and threat vigilance measures (ρ = .13; Kirk et al., 2021). The former measure (Hospital Anxiety and Depression Scale; Zigmond & Snaith, 1983) summates multiple dimensions of the symptomatology underlying anxiety disorders (e.g., worry/rumination, somatic sensations, panic attacks) which may each engage different internal processes (Baumeister et al., 2007; Watson et al., 2017). By having a self‐report measure which averages across multiple latent dimensions, there is likely reduced sensitivity to detecting specific internal processes.

“Higher‐order” symptoms (e.g., worry) vs more “fundamental” processes (e.g., threat vigilance) have been traditionally discussed in the context of relying more so on cortical vs subcortical structures respectively (Paulesu et al., 2010; Somerville et al., 2010). This is one interpretation of why effects on subcortical connectivity were not apparent for our self‐report measure. However, given recent evolutionary change of the human subcortex, this distinction may not be as clear as previously thought (Chin et al., 2022). Instead, these dimensions likely differentially engage cortico‐subcortical circuitry (Grupe & Nitschke, 2013; Kolobaric et al., 2021). Consistent with the small correlation between self‐report and behavior, it may also be that the self‐report measure is not a strong indicator of behavioral responding to threat. Therefore, this connection may also be specific for behavioral responses to threat and unrelated to a person's consciously aware feelings of anxiety (LeDoux & Pine, 2016). In line with recent calls (Moriarity et al., 2022), these findings further highlight the need for greater emphasis in anxiety research to investigate when/how measures of symptom subtypes, behavior, and brain function converge and diverge. We recommend future work to expand the present analytical framework to better identify specific phenotypic variation by assessing item‐level self‐report scores (which were unavailable in the present study) as well as additional self‐report scales assessing different aspects of anxiety.

We have provided evidence that a behavioral index of threat vigilance may be a key process underlying chronic engagement of amygdala‐periaqueductal gray circuitry. However, the use of human fMRI provides us with only one perspective for clarifying the function of this circuitry in humans. Due to the inherent associations between the BOLD signal and anxiety with autonomic functioning (Hu et al., 2016; Iacovella & Hasson, 2011), fMRI constrains our ability to draw causal conclusions as to mechanisms underlying this circuitry. In order to further delineate the psychophysiological processes driving this circuitry in humans and why it is associated with threat vigilance (e.g., are these signals *directly* or *indirectly* related to vigilance and/or autonomic regulation?), we encourage the use of modalities than enable stronger causal inferences. For instance, regional recordings and stimulation via intracranial electroencephalography may help further tease apart how these regions contribute to the perceptual, cognitive, and autonomic processes associated with anxiety (Parvizi & Castner, 2018).

In the present study, we took a theory‐driven approach for pre‐selecting regions of interest. This was done to study a variety of anxiety‐relevant connections (i.e., 45 edges) while minimizing the degree of statistical correction. However, this comes with inherent inferential constraints. First, as our approach did not use a whole‐brain parcellation, there may be other connections relevant to self‐reported anxiety and threat vigilance which were missed by taking this approach. Therefore, we cannot infer that regions with no significant connections in the present results would be the same when using a whole‐brain parcellation. Second, several of our regions were not defined on a subject‐specific basis, which may reduce sensitivity to related signals. Arguably, the lack of detected effects (particularly in cortical regions) could thus have arisen due to the use of standard‐space masks. In the context of our hypothesis‐testing however, we do note that our dmPFC mask was sufficient for capturing anxiety‐relevant processes in the same subjects during movie‐watching (Kirk et al., 2021). We also noted effects in subcortical regions such as the BNST and periaqueductal gray. These masks were based on previous manual tracings in MNI space (on *N* = 10 and *N* = 53 subjects respectively). As these regions are notably small, this risked missing relevant signals due to minor misalignments between subjects. We therefore dilated and eroded these masks to create better overlap between subjects. However, as these regions neighbor other small, subcortical structures, white matter, and ventricles, this procedure risked bringing in noise and unrelated signals.

Moreover, the dataset was collected using a 3 Tesla magnet with relatively large voxels (3 × 3 × 4.44 mm) which—compared to 7 Tesla scanning—may not be as spatially precise for the small, subcortical regions defined in the present study (Huggins et al., 2021). Lastly, although we describe an absence of stimuli during scanning, we highlight that resting‐state scanning may not be a passive state. Rest can be considered a task in and of itself, with different effects across populations, and thus may not offer a completely neutral backdrop for studying intrinsic connections (Finn, 2021). Understanding this, our inferences regarding these structures are of course tentative and research using more refined spatial resolutions, other neuroimaging modalities, and subject‐specific definitions is needed.

## 5 CONCLUSION

The present study aimed to investigate whether an association was present between individual differences in anxiety and amygdala‐prefrontal connectivity while at rest; we did not observe such a relationship. We suggest this circuitry may only be engaged in response to anxiogenic stimuli and thus individual differences only emerge under such conditions. On the other hand, we noted a relationship between a behavioral measure of attentional bias to threat (“threat vigilance”) and amygdala‐periaqueductal connectivity. Much of the prior literature has discussed the role of this subcortical circuitry in responding to threatening cues. Here, we provide evidence that this may be chronically engaged, irrespective of anxiogenic features of the environment. Moreover, why this was observed for our threat vigilance measure, but not self‐report, we argue is due to the functional role of this circuitry in more fundamental processes related to threat vigilance. Future research using higher magnet strengths and other imaging modalities will likely prove fruitful for elucidating precise contributions of this subcortical circuitry to anxiety‐relevant processes.

## DISCLOSURE STATEMENT

O.J.R is funded by an MRC senior fellowship partially in collaboration with Cambridge Cognition and he is running an investigator‐initiated trial with medication donated by Lundbeck. He also holds an MRC‐Proximity to discovery award with Roche for work regarding work on heart rate variability and anxiety. He has also completed consultancy work for Peak, IESO digital health and Neumora. He is on the committee for the British Association of Psychopharmacology. P.A.K and A.J.H report no biomedical financial interests or potential competing interests.

## Supporting information


**Table S1** Mediation analyses.

## Data Availability

Experiment code and data derivatives are available at the Open Science Foundation (https://osf.io/cfdq7/). Raw data are available on request from the Cam‐CAN website (https://camcan‐archive.mrc‐cbu.cam.ac.uk/dataaccess/).
