# Supplementary material for: Threat vigilance and intrinsic amygdala connectivity
Source: Hum Brain Mapp. 2022 Apr 1;43(10):3283–92. doi: 10.1002/hbm.25851 (PMC9188965; doi:10.1002/hbm.25851)
Supplement: Supplementary file 1 — Table S1 Mediation analyses. [file HBM-43-3283-s001.docx]

**Supplementary Materials**

**Threat Vigilance and Intrinsic Amygdala Connectivity**

**By Peter A. Kirk, Avram, J. Holmes, & Oliver J. Robinson**

**Supplement 1. Mediation analysis**

To supplement planned analyses, we conducted mediation analyses using Pingouin’s mediation_analysis functionality. We tested both the effect of self-reported anxiety mediating the relationship between connectivity and threat vigilance, as well as the effect of threat vigilance mediating the relationship between connectivity and self-reported anxiety (with age, sex, and movement as covariates for both tests). We tested this across three connections: left amygdala-dmPFC, right amygdala-dmPFC, and left amygdala-periaqueductal gray. The first two represented our hypothesized connections, the latter reflecting our significant finding from planned exploratory analyses. We note no significant effects (supplemental table 1). We do highlight here that ***the assumptions regarding these tests were not the same as those conducted in planned analyses*** as these assumed linearity, whereas partial spearman correlations in the manuscript tested for monotonic relationships.

| Supplemental table 1. Mediation analyses. | | | | |
| --- | --- | --- | --- | --- |
| X | Y | Mediator | Direct effect: Coefficient (p-value) | Indirect effect: Coefficient (p-value) |
| Left amygdala x dmPFC | Self-reported anxiety | Threat vigilance | .33 (*.66*) | .02 (*.70*) |
|  | Threat vigilance | Self-reported anxiety | -.02 (*.79*) | .00 (*.85*) |
| Right amygdala x dmPFC | Self-reported anxiety | Threat vigilance | .94 (*.19*) | .00 (*.96*) |
|  | Threat vigilance | Self-reported anxiety | .01 (*.92*) | -.01 (*.54*) |
| Left amygdala x periaqueductal gray | Self-reported anxiety | Threat vigilance | .21 (*.78*) | -.10 (*.50*) |
|  | Threat vigilance | Self-reported anxiety | .14 (*.05*) | .00 (*.84*) |
